# Mast Cell and Basophil Granule Proteases - *In Vivo* Targets and Function

**DOI:** 10.3389/fimmu.2022.918305

**Published:** 2022-07-05

**Authors:** Lars Hellman, Srinivas Akula, Zhirong Fu, Sara Wernersson

**Affiliations:** ^1^Department of Cell and Molecular Biology, Uppsala University, The Biomedical Center, Uppsala, Sweden; ^2^Department of Anatomy, Physiology, and Biochemistry, Swedish University of Agricultural Sciences, Uppsala, Sweden

**Keywords:** mast cell, tryptase, chymase, serine protease, CPA3

## Abstract

Proteases are stored in very large amounts within abundant cytoplasmic granules of mast cells (MCs), and in lower amounts in basophils. These proteases are stored in their active form in complex with negatively charged proteoglycans, such as heparin and chondroitin sulfate, ready for rapid release upon MC and basophil activation. The absolute majority of these proteases belong to the large family of chymotrypsin related serine proteases. Three such enzymes are found in human MCs, a chymotryptic enzyme, the chymase, a tryptic enzyme, the tryptase and cathepsin G. Cathepsin G has in primates both chymase and tryptase activity. MCs also express a MC specific exopeptidase, carboxypeptidase A3 (CPA3). The targets and thereby the functions of these enzymes have for many years been the major question of the field. However, the fact that some of these enzymes have a relatively broad specificity has made it difficult to obtain reliable information about the biologically most important targets for these enzymes. Under optimal conditions they may cleave a relatively large number of potential targets. Three of these enzymes, the chymase, the tryptase and CPA3, have been shown to inactivate several venoms from snakes, scorpions, bees and Gila monster. The chymase has also been shown to cleave several connective tissue components and thereby to be an important player in connective tissue homeostasis. This enzyme can also generate angiotensin II (Ang II) by cleavage of Ang I and have thereby a role in blood pressure regulation. It also display anticoagulant activity by cleaving fibrinogen and thrombin. A regulatory function on excessive T_H_2 immunity has also been observed for both the chymase and the tryptase by cleavage of a highly selective set of cytokines and chemokines. The chymase also appear to have a protective role against ectoparasites such as ticks, mosquitos and leeches by the cleavage of their anticoagulant proteins. We here review the data that has accumulated concerning the potential *in vivo* functions of these enzymes and we discuss how this information sheds new light on the role of MCs and basophils in health and disease.

## Introduction

Mast cells (MCs) are tissue resident cells that are found primarily in regions of the body in contact with the external world, such as the skin, the tongue, the small intestine, the lungs and in connective tissues surrounding different organs (1). They are also often found close to blood vessels and nerves where they act as sentinel cells, sensing the environment, signaling to other immune cells, and opening the tissues for influx of antibodies, complement and inflammatory cells. To perform these tasks they store massive amounts of physiologically acting substances in cytoplasmic granules, ready for rapid release upon activation of the cell. In these granules we find histamine, heparin and several proteases (2–10). The proteases, which are present in very large amounts can account for up to 35% of the total cellular protein of the MC, and they are stored in their active form in tight complex with negatively charged proteoglycans such as heparin and chondroitin sulfate (3, 4, 9, 11, 12). The absolute majority of these proteases belong to the chymotrypsin related serine protease family of endopeptidases (13). MCs primarily express chymases and tryptases with chymotryptic or tryptic primary cleavage specificities, respectively. The chymase is MC specific and the tryptase is expressed in both MC and basophils. MCs also express a carboxypeptidase named CPA3 that is restricted in its expression to MCs and basophils (14–17). This A/B type exopeptidase belongs to the M14 metallo-carboxypeptidase family. Upon activation MCs also produce and secrete potent lipid mediators, primarily leukotriene C4 (LTC4), and prostaglandin D2 (PGD2) (10, 18–20). Leukotriene B4 (LTB4) is also be produced by MC. However the major producer of this leukotriene seems to be neutrophils and monocytes/macrophages (21).

**Figure 1:**
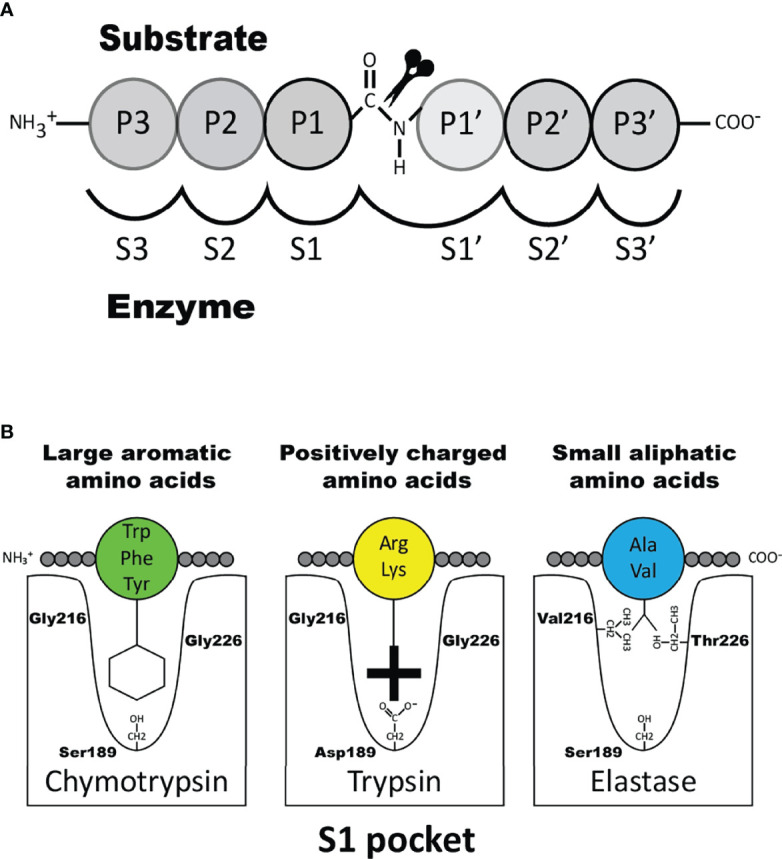
Nomenclature of the amino acids surrounding the cleaved peptide bond and the amino acids forming the active site pocket. Panel **A** shows the amino acids N-terminal from the cleaved bond are termed P1 (where cleavage occurs, depicted by scissors), P2, P3 etc. Amino acids C-terminal of the cleaved bond are termed P1’ (adjacent to P1), P2’, P3’ etc. The corresponding interacting sub-sites in the enzyme are denoted with S. Panel **B** shows the S1 pocket, which is important in determining the primary specificity of the chymotrypsin family. The important residues are shown, which determine chymotrypsin-, trypsin- or elastase-like specificity. Three residues corresponding to positions 189, 216 and 226 in bovine pancreatic chymotrypsinogen has been found to be the amino acids forming the major part of this pocket and thereby giving the primary specificity of the enzyme (24).

The transcripts for the granule proteases are the most highly expressed transcripts in the mature MC where the expression levels can be in the range of several percent of the total transcriptome (1, 16, 22). In addition to MC tryptase and chymase, serine proteases belonging to the same family of trypsin/chymotrypsin related serine proteases have in mammals also been identified in basophils, neutrophils, cytotoxic T cells and natural killer (NK) cells but not in B cells, macrophages, red blood cells, platelets or dendritic cells and only at relatively low levels in eosinophils. A very broad spectrum of primary cleavage specificities has been observed for these hematopoietic serine proteases, including chymase, tryptase, asp-ase, elastase and met-ase specificities, which highlights the large flexibility in their active site (23). It is the amino acids of active site pocket that determines the primary specificity and it is primarily three residues that are responsible for this specificity, i.e. the residues 189, 216 and 226, according to the numbering in bovine pancreatic chymotrypsinogen (**Figure 1**) (24).

The genes encoding these hematopoietic serine proteases are organized in four different loci, i.e. the MC chymase locus, the MC tryptase locus, the T cell tryptase locus and the met-ase locus (25, 26). In mammals, these loci are often located on four different chromosomes, indicating that some of them may originate from whole genome duplications, so called tetraploidizations. Two such tertraploidizations have most likely occurred during early vertebrate evolution, which indicate that the original locus was present before these evolutionary important events (27). Interestingly, only one of these loci is present in cartilaginous fish, the T cell tryptase locus, which also is named the granzyme A/K locus. Not any of these loci is present in jawless fish, including lamprey and hagfish, indicating that they appear at the base of jawed vertebrates (25).

**Figure 2:**
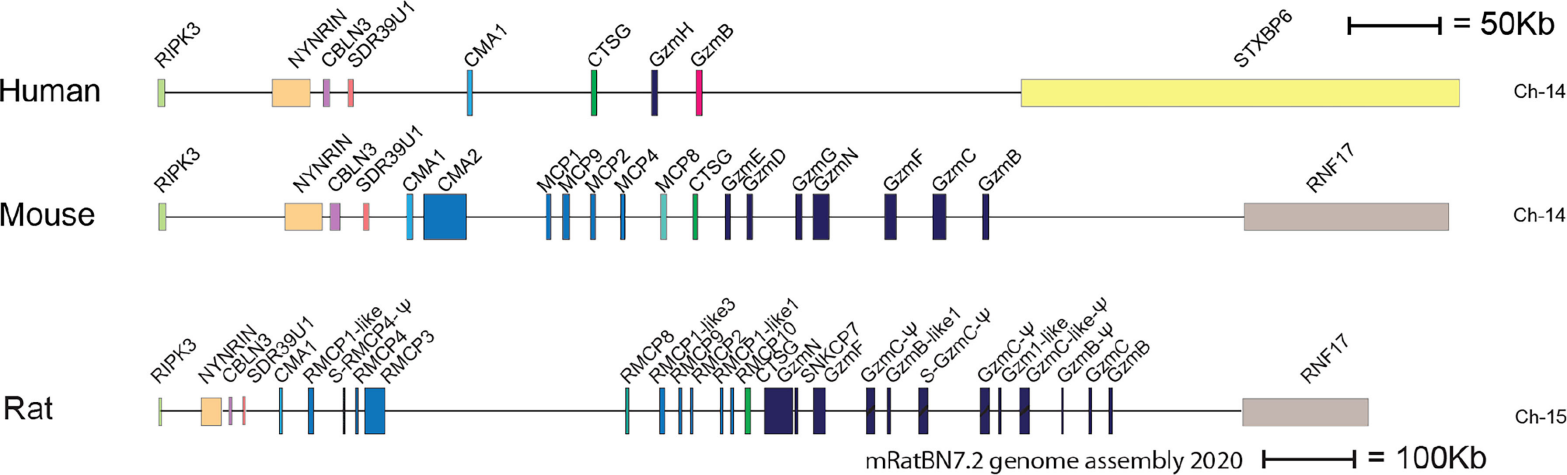
The chymase locus of three mammalian species. An in-scale figure of the chymase locus in three mammalian species; human, mouse and rat. The genes are color coded. Granzymes are shown in dark blue, α-chymases in light blue and β-chymases as blue with a darker tint. mMCP-8-related genes are in cyan, duodenases in red and cathepsin G in green. The general scale bar of 50 kB is valid for most of the genes. However, the rat locus is much larger in size why we have reduced the size to half. For rat we have therefore introduced a 100 kB size marker.

Two major subtypes of MCs have been identified in mammals, connective tissue MCs (CTMCs) and mucosal MCs (MMCs) (28–30). They have been named primary based on their tissue distribution. In humans and rodents CTMCs are primarily located in connective tissue of the skin, the tongue and in connective tissue surrounding different organs, whereas MMCs are found in the intestinal mucosa of the duodenum and in primates also in the lung (1, 31, 32). These MC subtypes express different sets of the MC specific proteases. In humans the MMCs are also named MC_T_, because they only express the tryptase, whereas the human CTMCs, also named MC_TC_ express four proteases, the tryptase, the chymase, cathepsin G and CPA3 (30). Interestingly, relatively large differences in the number of these proteases and their tissue specificity have been observed between species. As mentioned above human MCs primarily express four such proteases, the chymase, the tryptase, cathepsin G and CPA3 (33, 34). In marked contrast, mouse and rat have a relatively large number of such proteases. Mouse CTMCs express mMCP-5, the evolutionary counterpart of the human chymase, a second chymase named mMCP-4, two tryptases, named mMCP-6 and -7 and the carboxypeptidase CPA3 (1). The name for the human chymase gene and mMCP-5 in the locus annotation is Cma1. Cma1 is also classified as the α-chymase. In rodents a second subfamily of chymases has been identified, a subfamily named ß-chymases. These ß-chymases most likely originate from a relatively early gene duplication of the α-chymase gene. This initial duplication was then most likely followed by several successive duplications. In the mouse chymase locus there are five active such ß-chymases: mMCP-1, mMCP-2, mMCP-4, mMCP-9 and mMCP-10 (**Figure 2**). Mouse CTMCs express one of them, the mMCP-4. Mouse MMCs only express chymases, mMCP-1 and mMCP-2, and no tryptase or CPA3. To this should be added that the evolutionary counterpart of human chymase in mice, the mMCP-5, is per definition no longer a chymase since it lacks chymase activity. During evolution, the origin of mMCP-5 has by mutations of the amino acids surrounding the substrate binding pocket changed its primary specificity from being a chymase to now only accepting smaller aliphatic amino acids and thereby becoming an elastase (**Figure 1**) (35, 36). Interestingly, when the α-chymase mMCP-5, became an elastase, the ß-chymase mMCP-4 seems to have taken over the role of the α-chymase. mMCP-4 has been found to have an extended cleavage specificity almost identical to the human MC chymase (37). A similar expansion of the number of chymases is also seen in the rat (**Figure 2**). However, here the number of ß-chymases is even higher. Rats have seven ß-chymases: rat (r)MCP-1, rMCP-2, rMCP-3, rMCP-4, rMCP-1-like 1 (also named the vascular chymase (Vch)), rMCP-1 like 2 and rMCP-1 like 3 (**Figure 2**) (25).

This massive increase in protease genes in rodent is primarily seen in one of the four loci encoding the hematopoietic serine proteases, i.e. in the chymase locus (**Figure 2**) (25, 26). This locus encodes in humans only four genes, the MC chymase, cathepsin G and two T cell expressed granzymes, the granzymes B and H. In marked contrast, this locus encodes 15 functional protease genes in mouse and as many as 18 such genes in rat (**Figure 2**). Why rodents seem to need so many more of these genes compared to primates is still an open but very interesting question.

Basophils also express some of these proteases but store them at lower levels compared to MCs. Human basophils express the tryptase and CPA3, whereas mouse basophils express the basophil specific proteases mMCP-8 and mMCP-11 together with CPA3 (34, 38–41).

A number of very diverse functions have been identified for the different hematopoietic serine proteases, including apoptosis induction, blood pressure regulation, inactivation of insect and snake venoms, killing of bacteria and fungi, mobilization or degradation of cytokines and the degradation of connective tissue components (23, 42–47). These MC proteases may act both locally and systemically. However, in the circulation the presence of large amounts of protease inhibitors results in that the chymase rapidly become inactivated upon entering the circulation. The tryptase tetramers do relatively rapidly form monomers after entering the circulation and the monomers are inactive at physiological pH. The activity of these proteases is therefore primarily confined to the area close to the mast cells within the tissue and in there in macromolecular complex with the proteoglycans, and only to a minor extent in the circulation. The situation for the two tryptases in the mouse, mMCP-6 and mMCP-7, is slightly different. mMCP-6 seems to stay at the site and is not found in the circulation, whereas active tetramers of mMCP-7 are found in relatively high amounts in plasma of mice, but is gone from the circulation 4 hours after MC activation (48). In this review we will try to summarize the evidence that has accumulated on the biological targets for the very abundant MC enzymes and the less abundant basophil proteases and what this new knowledge can tell us about their role in MC and basophil biology. We will first look at the targets for the chymases, where most data have accumulated, followed by the tryptases and CPA3 and finally the basophil specific proteases. By applying an evolutionary approach we aim to identify potential targets that have been conserved among the majority of mammals which would strongly support their evolutionary importance as targets for particular enzymes.

## *In Vivo* Targets for the MC Chymases

### Angiotensin I and The Regulation of Blood Pressure

**Figure 3:**
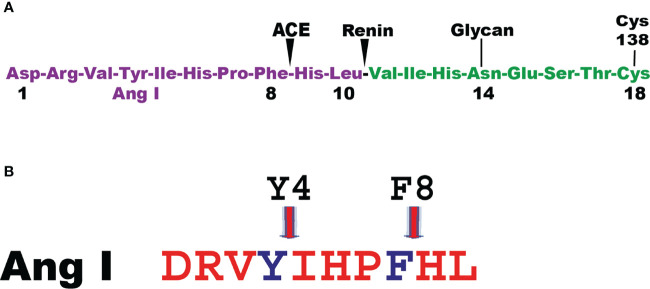
Angiotensin cleavage. Panel **A** shows the N-terminal end of angiotensinogen including the 10 amino acid and the cleavage sites in angiotensinogen for renin and for the angiotensin converting enzyme (ACE). Panel **B** shows the Ang I peptide with the cleavage site for the majority of MC chymase at Phe 8 generating Ang II and for a few additional MC chymases from some species where their chymases also degrade Ang II by cleavage at Tyr 4.

Angiotensin I (Ang I) is one of the first potential targets to be identified for the human MC chymase. Ang I is a 10 amino acid inactive peptide originating from cleavage of an elongated N terminus of angiotensinogen by the aspartyl protease renin (49) (**Figure 3**). Angiotensinogen is a member of the serpin family of protease inhibitors mainly produced by the liver but mRNA is also found in many other organs (50, 51). However, no inhibitory activity on proteases has been detected for angiotensinogen (49). Cleavage of Ang I by the human chymase at residue Phe8 results in the eight amino acid peptide Ang II. Ang II, but not Ang I, is a ligand for the G-protein coupled angiotensin receptors 1 and 2 (AT1 and AT2) (52). Most of the classical effects by Ang II is mediated by AT1, involving induction of blood vessel contraction, aldosterone release from the adrenal zona glomerulosa, salt retention in the renal proximal tubules and stimulation of the sympathetic nervous system thereby resulting in an increase in blood pressure (52). In some mammals the MC chymase also cleaves after Tyr4 in Ang I and thereby also degrade the peptide (**Figure 3**) (53, 54). The major Ang II generating enzyme in the blood has classically been considered to be the angiotensin converting enzyme (ACE) (**Figure 3**) (52). However, Ang I conversion in the tissues may to a larger extent be dependent on other enzymes including the MC chymases (55). Human, macaque and dog MC chymases, and mouse mMCP-4 seem to almost exclusively generate Ang II from Ang I by cleavage at Phe8 (53, 54). However, the major rat CTMC chymase, rMCP-1, seems to efficiently cleave at both Tyr4 and Phe8 and thereby degrade Ang I (**Figure 3**) (53, 54). A similar situation has been observed for golden hamster, and opossum chymases indicating that not all mammals have MC chymases with Ang I converting function (54). No studies have so far been performed to look for other potential enzymes in these species, which could have this function, except for in the rat (54). In the rat one of the ß-chymases have changed tissue specificity and is now no longer considered to be expressed in MCs, but instead in vascular muscle cells, therefore it has been named rat vascular chymase (Vch) (54, 56, 57). This protease was previously thought to have taken over the role of Ang I converting enzyme from rMCP-1, which also degrades Ang I by cleavage at Tyr4 (53, 56). However, when we recently analyzed the Ang I converting activity of recombinant Vch we found that it was a very poor Ang I converter (54). The question therefore remains if other enzymes in rat, hamster and opossum is responsible for Ang I conversion in these species or if they have alternative strategies to cope with blood pressure drop upon massive MC activation.

To have the MC enzymes involved in blood pressure regulation makes biologically a lot of sense as a massive release of histamine, leukotriene C4 and prostaglandin D2 from MCs upon antigen triggering result in increased blood vessel permeability and outflow of plasma from the vessels leading to a drop in blood pressure (58). In such scenario, the generation of Ang II by MC chymase could be essential for survival to counteract a massive drop in blood pressure that could result in unconsciousness, and severe injuries after fainting.

### Matrix Components

The amount of proteolytic enzymes stored in the CTMCs is remarkable. In spite of the fact that the granules already are filled with these enzymes, MCs still seem to constantly synthesize these enzymes. Their mRNA levels are namely exceptionally high even in resting tissue CTMCs (1, 16, 22). It has been proposed that these tissue resident MCs secrete small amounts of these proteases by piece meal degranulation, possibly constantly as a part of normal tissue homeostasis. The human, mouse, macaque, dog and opossum MC chymases have been shown to efficiently cleave fibronectin and at least the human chymase also seems to activate pro-collagenases indicating that they play an active role in connective tissue turnover (43, 44, 47, 59). Findings from mMCP-4 knock out mice support the role of the MC chymase in connective tissue homeostasis where the connective tissue in these mice increase in thickness with increasing age indicating a reduced turnover of the connective tissue components (59). A similar phenomenon has been observed in mice lacking macrophage colony stimulating factor (M-CSF) where these mice lack or have very low numbers of osteoclasts, a type of bone macrophages. These mice develop osteopetrosis with very dense bone that become brittle due to that their bone matrix is not being degraded and resynthesized, which results in small cracks in the bone that are not being sealed (60). So at least two different immune cells may have an important role in general tissue homeostasis, i.e. MCs of connective tissue and macrophages of both bone and connective tissue. Mouse peritoneal macrophages also express high levels of several connective tissue components including fibronectin, proteoglycan 4, syndecan 3 and extracellular matrix protein 1 indicating that they are major players in connective tissue homeostasis (61).

### Regulating Intestinal Permeability

**Figure 4:**
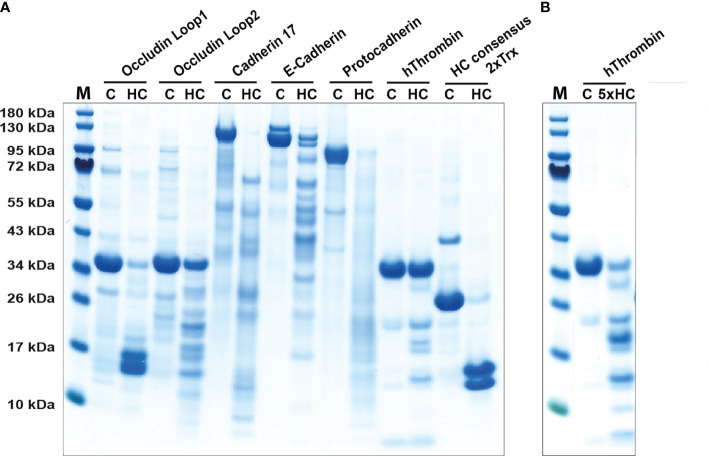
Cleavage of adhesion molecules and thrombin by the human chymase. An SDS-PAGE analysis of a cleavage analysis of a panel of different cell adhesion molecules and of the coagulation factor thrombin by the human MC chymase. C marks the target protein without addition of the chymase, as control, and HC marks the cleavage of the target molecule by the human chymase. As a control for the activity of the human chymase is also a recombinant 2xTrx substrate included, where an optimal cleavage site for this enzyme is inserted between two copies of the *E. coli* redox protein thioredoxin (43). In panel **(A)** forty ng of HC was used in each of the cleavage reactions. In panel **(B)** which is a second cleavage reaction included of thrombin, there we used five times higher amount, or 200 ng, of the human chymase.

During parasite infections MMCs increase in numbers quite dramatically in the intestinal mucosa peaking between one and two weeks after a worm infestation (29, 62). The numbers can increase by 10-50 fold and the majority of these new MCs are located just below the epithelial layer of the intestinal mucosa of the duodenum (29, 62, 63). Injection of the rat MMC-specific protease rMCP-2 has been shown to rapidly affect the permeability of the mucosal layer resulting in outflow of antibodies, complement components and also inflammatory cells, including eosinophils and neutrophils (64, 65). By analyzing the cleavage of a panel of cell adhesion molecules expressed in the intestinal mucosa of rats we found that rMCP-2 effectively cleave several of these components including protocadherin, E-cadherin, cadherin-17 and occludin (66). Interestingly, human MMCs lack chymases, so the question is if primates have alternative strategies to increase intestinal permeability. Injection of rMCP-2 intravenously, not only locally produced, has been shown to result in this increase in intestinal permeability indicating that also chymase originating from CTMC could have this effect. We therefore decided to test the ability of human chymase to cleave the same set of cell adhesion molecules that we used to analyze the activity of rMCP-2. We can here show that the human chymase actually is even more active against these cell adhesion molecules than the MMC-specific rMCP-2. Almost all of the tested targets, including protocadherin, E-cadherin, cadherin-17 and occludin were efficiently cleaved by the human chymase indicating that it may have a similar function as rMCP-2 (**Figure 4**), despite its absence in normal human MMCs.

### MCs in Immunity to Bacteria

The role of MCs in the defense against bacterial infection have been controversial due to the initially relatively poor animal models (67). The early MC deficient mouse models, primarily the W/W^v^ but also the W^sh^/W^sh^, that was based on mutations in the stem cell factor receptor, the c-kit, had indicated a clear involvement of MCs in bacterial defense. However, many of these effects and also the role of MCs in autoimmunity could not be confirmed when more stringent MC deficient mouse models became available (67). In these early MC-deficient models there were also reduced numbers and activity of neutrophils and also more general effects on hematopoiesis, which turned out to be the major cause of the reduced efficiency to combat bacterial infections in these mice. However, a clear role of MCs has later been seen with the new and improved MC-deficient animal model, including the CPA3-Cre, mMCP-5-Cre and Mcl-1fl/fl mice (67). These new models are considerably better than the kit dependent model, but also these new models have flaws. They also show reduced numbers of basophils (68).

One important example of the importance of MC in bacterial defense when using these new models is the defense against infection by *Escherichia coli* in the urinary bladder. In this type of infections the bacterial clearance has been well documented to be dependent on the mouse MC chymase (mMCP-4) (69). Upon infection by *E. coli*, MCs increase in numbers in the bladder wall and migrate towards the epithelial layer where they release their granules. The granule proteases do in turn cleave adhesion molecules and when mMCP-4 enter the epithelial cells it induces caspase 1 activation and pyroptotic cell death which results in the shedding of the infected epithelial layer of cells lining the bladder inner wall. The bacteria attached to the bladder wall are then flushed out of the bladder resulting in marked reduction in bacterial load and a faster recovery (69). The different MC proteases may have similar functions in the duodenum and the urinary bladder, by cleaving cell adhesion molecules resulting in increased permeability and/or in epithelial shedding. Epithelial shedding seems namely also to be a major defense strategy in the intestinal region during severe bacterial infections, like *Salmonella* infections (70). During severe *Salmonella* infections the infected cells of the epithelial layer are shed to remove the bacteria from the epithelial layer and to inhibit them from reaching deeper into the intestinal tissue (70). Although the involvement of MCs in this intestinal shedding seem very likely, this has to our knowledge not yet been documented. Notably, a recent study has demonstrated a role for both MCs and IgE in the clearance of bacterial infection in the ear of mice. The importance of MCs in this study was linked to protection against secondary lung infection by *Staphylococcus aureus* (71) and there were indications that the MC proteases participated in the defense (71). MC proteases probably also contribute to bacterial clearance by cleavage of many bacterial virulence factors. However, there are so far few documented examples of such potential targets of importance for the bacterial defense.

Many bacteria use adhesion to connective tissue components, primarily fibronectin as one way to attach and avoid being flushed out of a tissue. This attachment is achieved by the production of fibronectin binding proteins by bacteria (72). In at least one study the cleavage of fibronectin by the mouse chymase mMCP-4 have been shown to be of importance for inhibiting bacteria to colonize a tissue and thereby having a beneficial role in bacterial clearance (72).

### Regulating coagulation

Granules of rodent CTMCs contain large amounts of heparin and in primates both CTMCs and MMCs contain heparin (73). Heparin is a potent anticoagulant by the activation of anti-thrombin (74). The triggering of MCs results in the release of histamine, leukotrienes, prostaglandins, heparin and proteases. These mediators do in turn trigger the opening of blood vessels, which results in an influx into the tissue of antibodies, complement, inflammatory cells and blood coagulation components (58, 75). When clotting proteins come in contact with the tissue, coagulation is initiated to seal the leakage of the vessel. The potentially negative effect of coagulation is that the flow of immune components is blocked. To counteract this closure, MC heparin may limit activation of thrombin, one of the key enzymes in the clotting cascade. The MC chymase may also help in this inhibition of coagulation by actively inactivating thrombin by proteolytic cleavage. Cleavage of thrombin by human chymase has been shown previously (76). To further analyze the efficiency of this cleavage we have tested the *in vitro* cleavage of human thrombin with two different concentrations of the chymase (**Figure 4**). We found that considerably more of the enzyme was needed to cleave thrombin compared to the cell adhesion molecules, but still within the physiological range of the enzyme concentration. Hence, degradation of thrombin by the chymase may contribute to the inhibition of coagulation after opening of the vessels for immune mediators and cells. Chymase also efficiently cleave fibrinogen and this effect has also been evolutionary conserved as we have shown that both human and opossum chymase efficiently degrades fibrinogen (47). MCs can thereby have anticoagulant activity by at least three independent mechanisms, heparin and the inactivation of both thrombin and fibrinogen.

### Inactivation of Venoms

MCs are most abundant in tissues in close contact with the external world like the skin, the tongue and the intestinal mucosa. Venoms delivered by snake bites, scorpion stings or bee and wasp stings are generally appearing in the skin. Several elegant studies have shown the potent action of the different MC enzymes on these venoms resulting in their inactivation (42, 77–80). The venoms also act directly on the MCs by inducing degranulation (80).

The human MC chymase has been shown to cleave internally in endothelin and sarafotoxin 6B from the Israeli mole viper (78, 81). Interestingly several of these toxins mimic the action of endogenous activators. Some of these toxins show high sequence homology to endothelin, a potent endogenous regulator of blood pressure. Endothelin has been shown to be cleaved by both the human chymase and by CPA3 (81). Chymase cleaves internally in endothelin whereas CPA3 cleaves of a single amino acid in the C-terminal end of this peptide (77, 81). It is the C-terminal trimming, by the removal of the C-terminal tryptophan, that appears to have the major inhibiting effect on the biological activity of endothelin (81).

### Regulating Excessive T_H_2 Immunity

**Figure 5:**
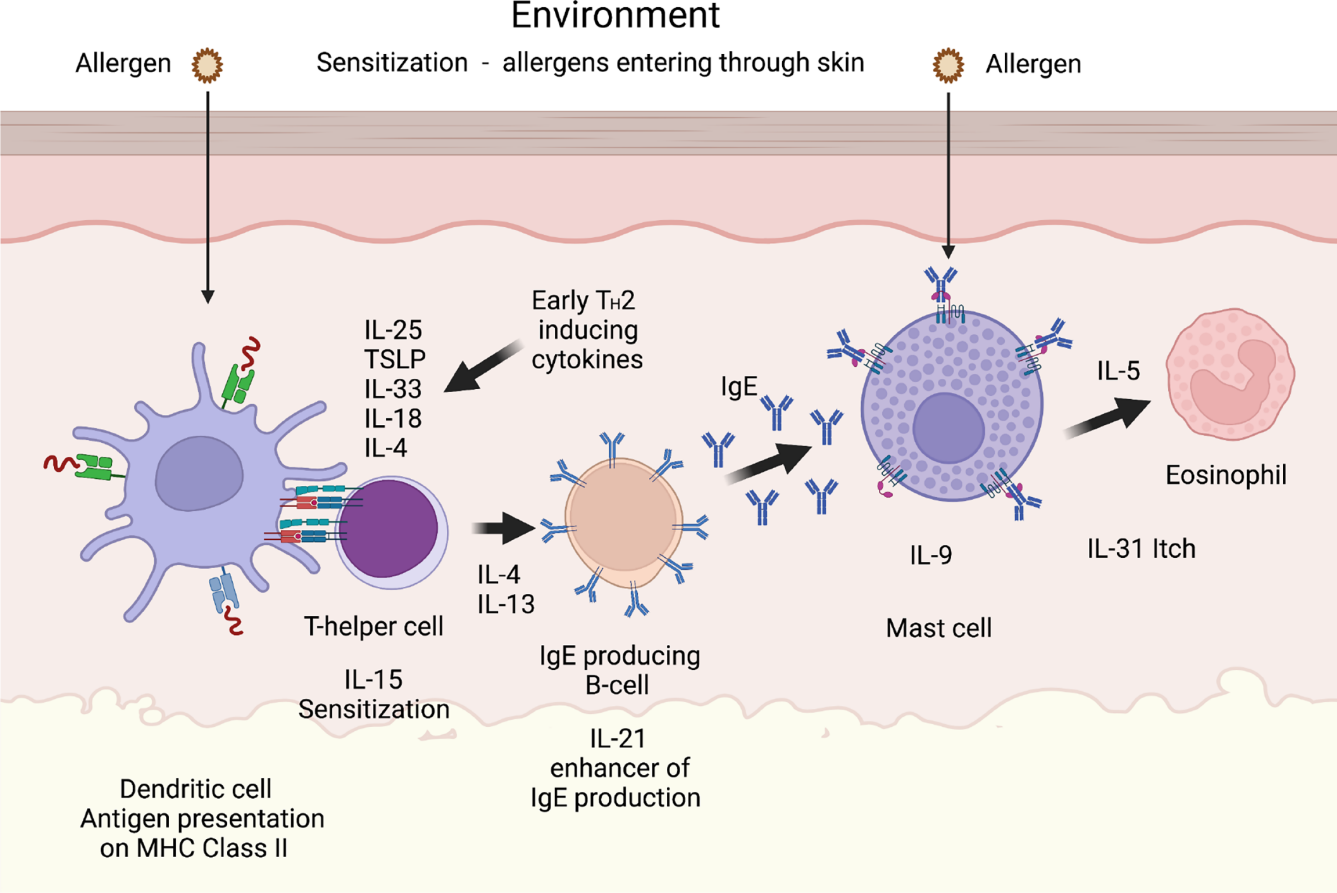
A T_H_2 immune response. A number of cytokines have been shown to be potent inducers of T_H_2 mediated immunity. The most well characterized are thymic stromal lymphopoietin (TSLP), IL-33, IL-18, IL-25 and IL-4. IL-18 has been shown to be potent inducers of T_H_2 mediated immunity when present alone and not in combination with IL-12. Interestingly when present together with IL-12, IL-18 acts instead as an enhancer of T_H_1 mediated immunity (85). IL-4 and IL-13 are the only two cytokines known to induce isotype switching in B cells to IgE (86). IL-5 is important for eosinophil infiltration, activation and proliferation, and IL-31 acts as an inducer of itch in skin of atopic dermatitis patients. IL-9 is, in mice, an inducer of mucosal mast cell differentiation (87). Both IL-15 and IL-21 has been found to have T_H_2 promoting activity as described in the text. Cleavage of the T_H_2 initiating early cytokines would most likely result in a dampening effect on T_H_2 mediated immunity.

The default pattern in immune regulation has been considered to be a humoral T_H_2 immunity, primarily involving B cells and immunoglobulins. This is an immune response not involving the activation of potentially harmful cytotoxic T cells. However, upon infection with intracellular parasites, including viruses and certain bacteria, like *Mycobacteria*, *Chlamydia* and *Listeria*, the immune system needs to activate a more potent response. However, this will also activate more potentially harmful players to locate and remove the infection including killing the infected cells by inducing apoptosis and subsequent phagocytosis of the infected cells. Antibodies are not sufficient to target infectious agents that hide inside our own cells. The important triggers of this cell mediated T_H_1 response are IL-12 and interferons, primarily IFN-α and IFN-ß. T_H_1 responses need to be very tightly regulated and very small amounts of IL-12 is apparently sufficient to activate this type of response. Attempts to regulate excessive T_H_2 immunity, which occur in allergic patients, by tilting immunity more towards T_H_1 immunity has not been successful, primarily due to the high potency of the IL-12. Strikingly, mice injected with 1 ug of IL-12 per day resulted in 100% death at day six due to the potency of the IL-12 (82). The question is if there also is a tight regulation of T_H_2 immunity and in that case what cell type would most likely be involved. MCs can produce a large number of cytokines, primarily T_H_2 cytokines, so they can potentially enhance an ongoing T_H_2 immunity (83). However, do they also have a role in controlling excessive T_H_2 immunity? In support of this notion, we found that MC chymase cleaves the T_H_2-cytokine IL-33 and exhibits a protective role in a mouse model of allergic asthma, as suggested by the augmented sensitization, airway inflammation and IL-33 levels in mice lacking the chymase mMCP-4 (84). To study the human chymase for its selectivity in degrading cytokines and chemokines we analyzed the activity of the human chymase on a panel of 55 cytokines and chemokines. To our surprise we found that this enzyme was exceptionally restrictive in its cleavage. Only three cytokines were efficiently cleaved by the human chymase, namely IL-18, IL-33 and IL-15 (46). Two of these, IL-18 and IL-33, are classical T_H_2 cytokines and the third, IL-15, has recently been shown to be a potent inducer of allergen sensitization, showing that also this cytokine has a potent T_H_2 inducing activity (**Figure 5**) (88, 89). This very high selectivity for a small set of T_H_2 cytokines, indicated an important role of the chymase in regulating excessive T_H_2 immunity (46). We later showed that also the tryptase has a very similar activity, which made the finding even more striking (47). These two MC-specific enzymes combined appear to have a very strong dampening effect on T_H_2 immunity. One major function of these abundant MC enzymes seems therefore to act as a potent negative feed-back loop on T_H_2 immunity (**Figure 5**). This should be kept in mind when trying to develop anti-allergy drugs by inhibiting the activity of the MC chymase and/or the tryptase. In contrast to what we observed for the human chymase, which did not cleave TNF-α, the mouse counterpart to the human chymase, mMCP-4, has been shown to be a major player in the inactivation of this cytokine *in vivo* (45). The targets for these mast cell enzymes can therefore differ significantly between species, a situation that needs to be kept in mind when using animal models to study the *in vivo* effect of these enzymes.

### Role In The Defense Against Ectoparasites

Basophils have been shown to accumulate at the site of tick infestation. However, this happens only after the second contact with the parasite and this accumulation is dependent on adaptive immunity, IgE and the high affinity IgE receptor (FcεRI) (90, 91). Mouse basophils express a protease that is specific for this cell type, the mMCP-8 (39, 62). To study the potential role of this enzyme in the defense against ticks and other ectoparasites we produced a tick anticoagulant protein (TAP) of *Ornithodoros moubata* and analyzed its sensitivity for cleavage by the basophil specific protease mMCP-8 and a panel of other hematopoietic serine proteases. In contrast to our expectations, we found that mMCP-8 had no effect on this tick anticoagulants. However, all the connective tissue MC chymases tested, including human-, dog-, rat-, hamster- and opossum chymases did very efficiently degrade this anticoagulant protein, indicating a potent role of the MC chymases in the defense against this ectoparasite (92). We then continued this analysis by analyzing the role of this panel of hematopoietic serine proteases on anticoagulants from two other ectoparasites, leeches and mosquitos. We tested their activity on the thrombin inhibitors hirudin from *Hirudum medicinalis* and on anophelin from the malaria vector *Anopheles gambiae*. Almost an identical pattern of cleavage was seen for these two anticoagulants indicating that the connective tissue MC proteases cleave the anticoagulants of a broad array of different ectoparasites (92). Interestingly, the mucosal MC proteases were in general inactive against these anticoagulant proteins indicating a high selectivity of these proteases in their target specificity (92). Connective tissue MCs are primarily located in the skin which is the primary site of infection for these ectoparasites, indicating a functional adaptation of the cleavage specificity to targets of importance for the particular type and tissue location of the MC. Ticks have been found to have the capacity to counteract this effect of chymases by producing protease inhibitors. One example of such an inhibitor has been found in the saliva of the tick *Ixodes ricinus*, the serpin IRS-2, which is a potent inhibitor of all the MC chymases tested (92, 93).

### Other Potential Targets for the MC Chymases

The potential targets for MC proteases as listed above, do most likely not include all the targets of importance for the physiological role of these enzymes. MCs and the different MC proteases have been shown to contribute, positively or negatively, to a number of conditions including arthritis, dermatitis, obesity, atherosclerosis, abdominal aortic aneurysm and cancer (94). This information has been obtained by studies of knock out mice for single and combinations of these proteases or with protease inhibitors. These studies have given a lot of valuable information concerning potential involvements of these proteases in the role of MCs *in vivo* (94). However, to our knowledge no more solid information concerning the most important *in vivo* targets for these proteases in the phenotypic changes that are observed in these studies have been reported. These models give primarily information concerning the importance of MCs or of individual MC proteases in a particular disease or organ function, but they seem to be less suitable for specifying the exact target for the proteases. Combinations of *in vivo* and *in vitro* studies are therefore essential for the fine mapping of the biological function of these proteases as they may have a very complex physiological role in immunity and tissue homeostasis. Surprisingly, the results from knock out mice and from inhibitor studies are also often contradictory as nicely shown in a recent review on the lessons from knockouts and inhibitors into the *in vivo* function of the MC chymase (94). Many of the chymase inhibitors are also affecting the activity of the related cathepsin G that is expressed in high levels in human MCs and the fact that most studies are performed in mice while the inhibitor specificity has been determined for the human chymase, is further complicating these analyses (33, 94). More detailed screenings for potential targets of importance for the biological effects observed are therefore needed before the picture of the role of the various proteases in the observed phenotypes can be obtained.

**Figure 6:**
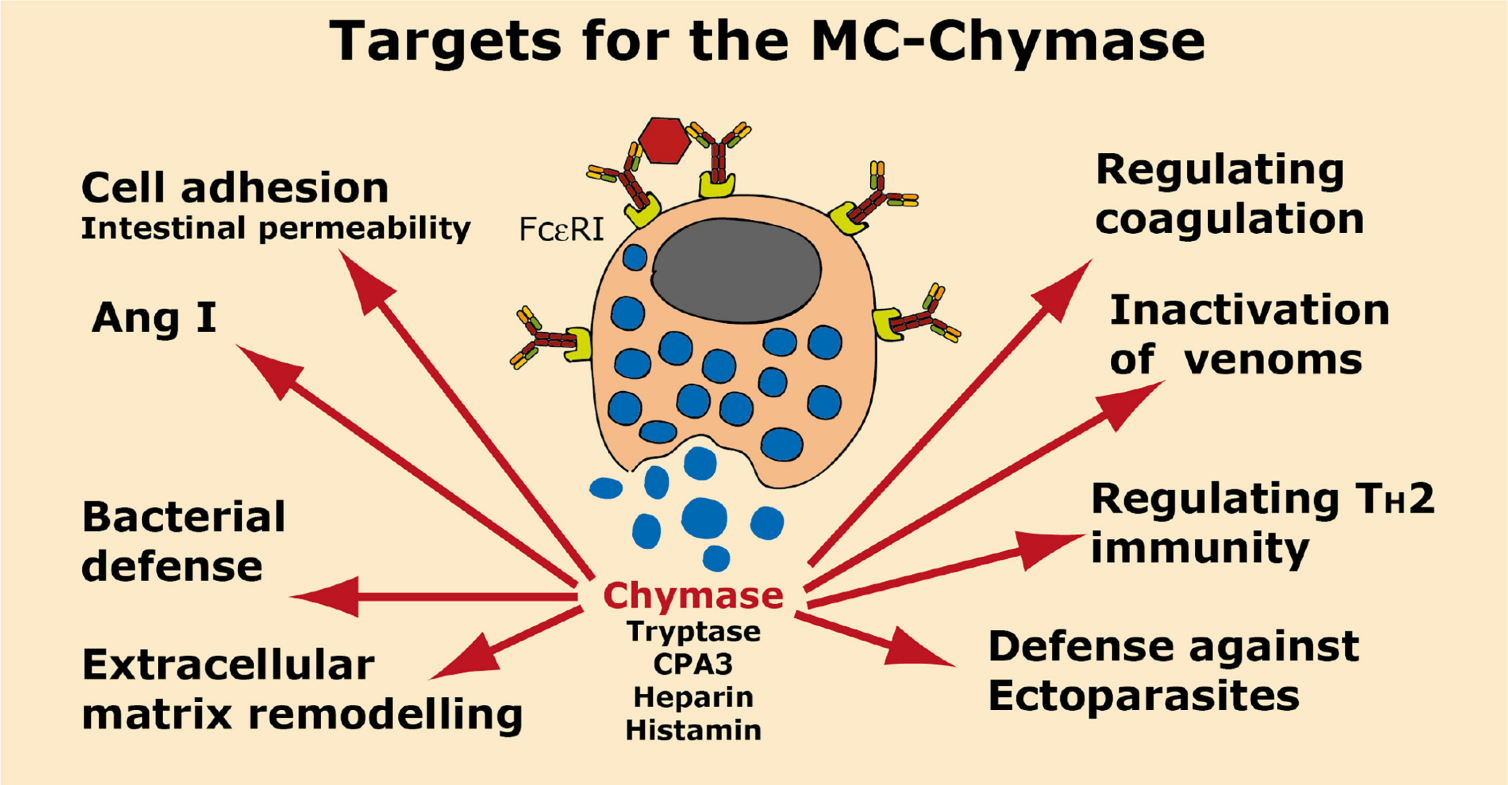
Targets for the human MC-Chymase. A number of potential targets for the human MC chymase is summarized.

A summary of the potential targets of the MC chymase is found in **Figure 6**.

## *In Vivo* Targets for the MC Tryptases

The target specificity for the tryptases are more restricted than for the chymases. One reason for this is their tetrameric structure. The active sites of tryptase are located in the center of the tetramer making them less accessible for larger substrates (95). The tryptase may also be active in its monomeric form, but this only at a pH lower than the normal physiological pH (96). During inflammatory conditions the pH may drop to 6.0-6.5 and then can the monomeric tryptases also show activity, and the monomers have then a less restrictive active site, potentially allowing larger substrates to enter that active site. However, due to that the most active form of the tryptase is in its tetrameric form most of the potential targets identified for tryptase has been peptides. However, as discussed above the human tryptase has also been shown to cleave a highly selective set of T_H_2 cytokines showing that not only peptides can serve as important targets (47). The human tryptase also trims the ends of fibrinogen, most likely resulting in the inability to form a fibrin clot, and that the tryptase may thereby cooperate with the chymase in controlling coagulation (47). The cleavage and activation of the protease activated receptor 2 (PAR-2) by human and mouse mast cell tryptases have been a controversial issue for several years, but seem now to be relatively well documented (95, 97, 98). The human MC tryptase has also been shown to be highly active in detoxifying six different snake venoms (80).

### Inactivation of Venoms

Evidence for a prominent role of tryptase in the defense against snake venoms has come from a study of the effect of human skin MC extract and purified human chymase, tryptase and CPA3 on the lethal effect of venom from six different snake species (80). In this study only tryptase, and not chymase or CPA3, had an effect on the survival rate of zebrafish embryos injected with venoms from a number of snake species, indicating that tryptase is the dominant player in the protection against snake venom (80). This study indicates that purified tryptase could be used as a potential treatment for snake bite envenoming. A few of the potential targets of the tryptase in these venoms were identified. One of them was L-amino acid oxidase, an enzyme that act on platelets and induce apoptosis and hemorrhagic effects, but can also induce apoptosis in a number of different cells, the second was a zinc metalloproteinase that induce capillary hemorrhage and myonecrosis (80).

### PAR-2 Cleavage

Very recently the role of the mouse mast cell tryptase, mMCP-6, in PAR-2 cleavage and its role in the induction of thymic stromal lymphopoietin (TSLP) expression in mouse skin keratinocytes have been demonstrated (97). In this study, keratinocytes from PAR-2 negative mice did not show this induction of TSLP strongly suggesting that PAR-2 is a target for the mast cell tryptases (97). These findings also suggest that the MC tryptase can be an important trigger of inflammatory cytokines by keratinocytes and possibly also other tissue cells. Interestingly in this study histamine was also found to induce several cytokines and chemokines, although not TSLP but instead several other cytokines and chemokines including IL-6, stem cell factor (SCF) and IL-8 (97).

PAR-2 is also an important component in the blood coagulation system and several coagulation proteases, including FXa and FVIIa, can activate PAR-2 (99).

### Histone Modification

The tryptase has been shown to leak out from the granules in small amounts and to enter the nucleus where it can cleave the core histones in their N-terminal tail indicating that the tryptase can have a regulatory role on transcription (100).

### Peptide Substrates for the MC Tryptases

A number of peptides have been identified as potential substrates for the MC tryptases including kininogen, vasoactive intestinal peptide (VIP), peptide histidine-methionine (PHM) and calcitonin gene-related peptide (CGRP) indicating that this MC enzyme can have a potent regulatory function in several tissues (95, 101–103). The degradation of several of these mediators of bronchodilation may lead to increased bronchial responsiveness and may at least partly explain the induction of bronchial hyper-reactivity by tryptase (95).

### Regulating Excessive T_H_2 Immunity

Due to the remarkable selectivity in the cleavage of cytokines and chemokines by the human chymase, we decided to continue the screening for potential similar activities of the other major granule serine protease of human MCs, the tryptase. The tryptase was found to be even more selective than the chymase. We detected cleavage of only two cytokines out of a panel of 69 different human cytokines and chemokines (47). The two cytokines cleaved by the human tryptase were two additional key T_H_2 cytokines, TSLP and IL-21 (**Figure 5**) (47). TSLP has been named a master regulator of T_H_2 immunity and IL-21 was recently identified as a potent enhancer of IgE synthesis (104, 105). When we combined the action by both the chymase and the tryptase we found a remarkable selectivity of cytokines cleaved by these two enzymes. All five cytokines cleaved by these two enzymes are key T_H_2 cytokines, either directly involved in the induction of a T_H_2 cytokine environment, or in sensitization or IgE production. This coordinated action by the two major human MC proteases is in our mind an exceptionally strong indication for a potent role of these two MC proteases and of the MC in controlling excessive T_H_2 immunity (**Figure 5**). We will therefore stress the potential danger with using tryptase and chymase inhibitors in the treatment of T_H_2 mediated inflammation including asthma, dermatitis and other MC-dependent diseases. The long-term effect may be the increase in T_H_2 mediated immunity, by increased sensitization to a broader panel of allergens and an exaggerated allergic inflammation. A clear indication for such an effect was observed in mice lacking the major MC chymase in mice, mMCP-4, which were found to develop exaggerated responses in two models of allergic airway inflammation (84, 106). The lack of both the chymase and the tryptase may here be even more pronounced. Analysis of double knock out mice for both mMCP-4 and mMCP-6, the counterpart of the human tryptase, would therefore be very interesting for its effects on allergen sensitization. However, similar to what was observed for the chymase with TNF-α, there are differences in the cytokines and chemokines cleaved by the MC tryptases in mouse and man. The human tryptase cleaves TSLP and IL-21. The corresponding enzyme in mouse, mMCP-6, does not cleave TSLP, but cleaves IL-21 and also IL- 9, IL-13 and IL-33, indicating that care should be taken when comparing data from human and mouse studies as the exact targets may differ (47).

Interestingly, the tryptases can thereby both induce the expression of certain cytokines, such as TSLP, by acting on PAR-2 on keratinocytes and at the same degrade this cytokine, indication a complex pattern of regulation of cytokines and chemokines by these enzymes.

**Figure 7:**
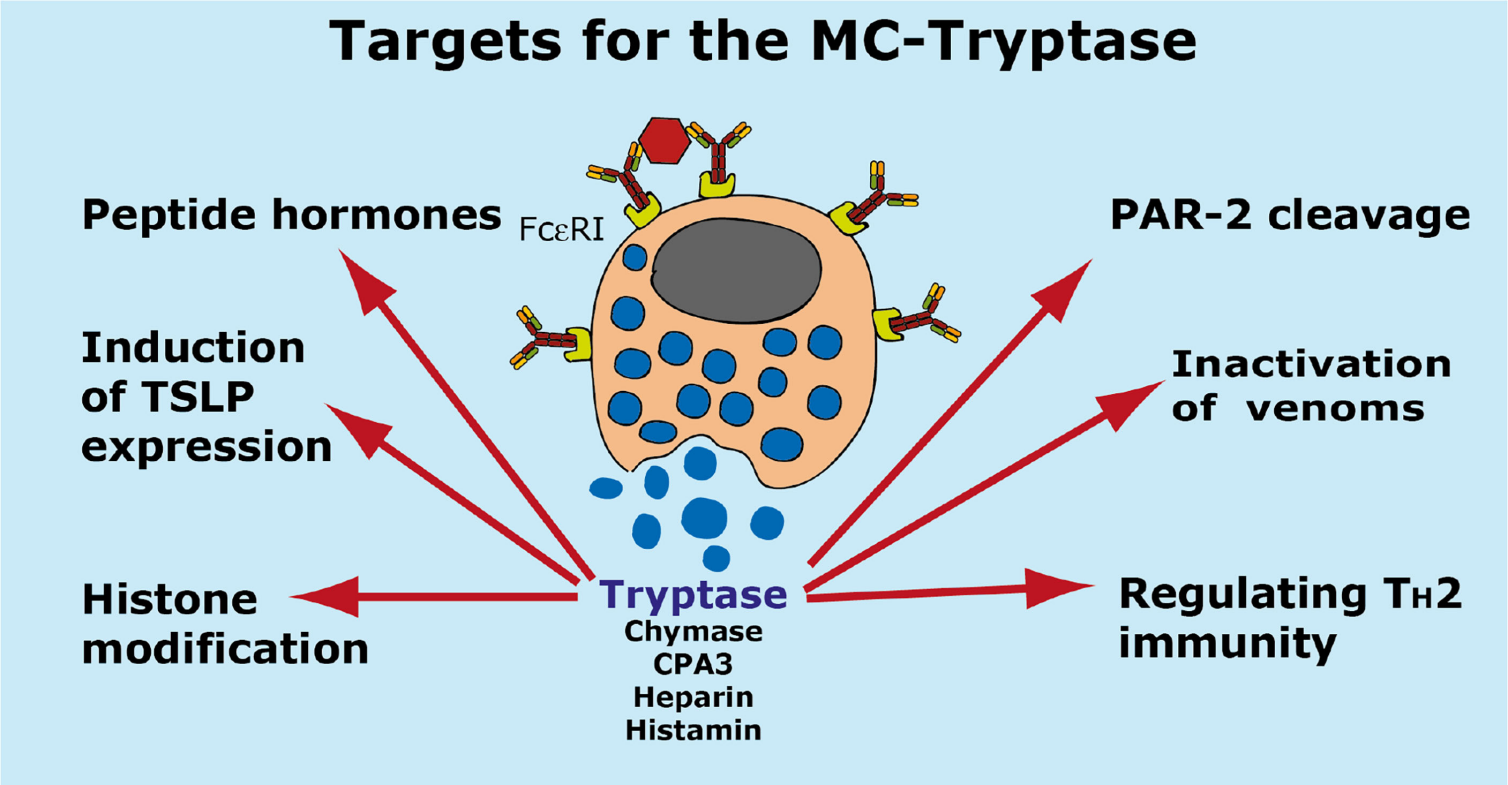
Targets for the human MC-Tryptase. A number of potential targets for the human MC tryptase is summarized.

A summary of the potential targets of the MC tryptase is found in **Figure 7**.

## *In Vivo* Targets for the MC and Basophil Expressed CPA3

The potential targets for the MC and basophil specific CPA3 have been even more difficult to determine compared to the tryptases due to that it is an exopeptidase and not an endopeptidase. A minor trimming of a C-terminal end of a protein is much more difficult to spot than an internal cleavage, which markedly change the molecular weight of the target. Removing one or a few amino acids of a large protein is often difficult to detect on a gel. The major difficulties in obtaining recombinant CPA3 is another major factor for the low number of potential *in vivo* targets identified for this enzyme. The mouse CPA3 apparently needs co-expression with mMCP-5 for the presence in granules indicating a complex transport through endoplasmic reticulum and Golgi for correct processing and storage (107–109). Production of active mouse and human CPA3 has therefore been very difficult and no active mouse or human recombinant CPA3 are presently available for more detailed studies of their potential *in vivo* targets. We have tried with both mammalian and insect cells without success.

The most well documented substrate for the CPA3 is so far endothelin, a potent vasoconstrictor, and the related snake venom component sarafotoxin (77, 81). Endothelins, which are secreted from vascular endothelium, are the most potent vasoconstrictors known (110). They act through two G-protein couplet receptors, ETA and ETB (110). Removing the C-terminal tryptophan results in the inactivation of the 21 amino acid endothelin peptide (81). A few additional peptide hormones have been identified as potential *in vivo* substrates for this enzyme including neurotensin, kinetensin, Leu-enkephalin, neuromedin N and Xenopsin (109, 111–114). However, no more direct functional studies of the *in vivo* effects of these latter targets have been performed as we can see from Pubmed.

Ang I and apolipoprotein B has also been identified as potential targets. CPA3 can remove the C-terminal Leu of Ang I to generate Ang 1-9 (**Figure 3**) (112). CPA3 can also trim apolipoprotein B, a low density lipoprotein (LDL) involved in cholesterol transport (115). This cleavage can promote LDL fusion and thereby plaque formation.

The collected information concerning the biology of CPA3 is thereby relatively limited why additional studies are needed for a better understanding of the role of CPA3 in MC biology and general physiology. However, the difficulty in obtaining recombinant active enzyme is a major hurdle to overcome before additional *in vitro* and *in vivo* studies of this abundant MC and basophil enzyme can be performed. A possible role for CPA3 in regulating T_H_2 immunity has not been demonstrated although CPA3 expression has been linked to T_H_2 type of asthma in humans (116, 117). We recently found a dampening effect of a CPA inhibitor in a mouse model of allergic asthma, however, the individual role of CPA3 compared to other types of CPAs in this model has not been determined (118).

**Figure 8:**
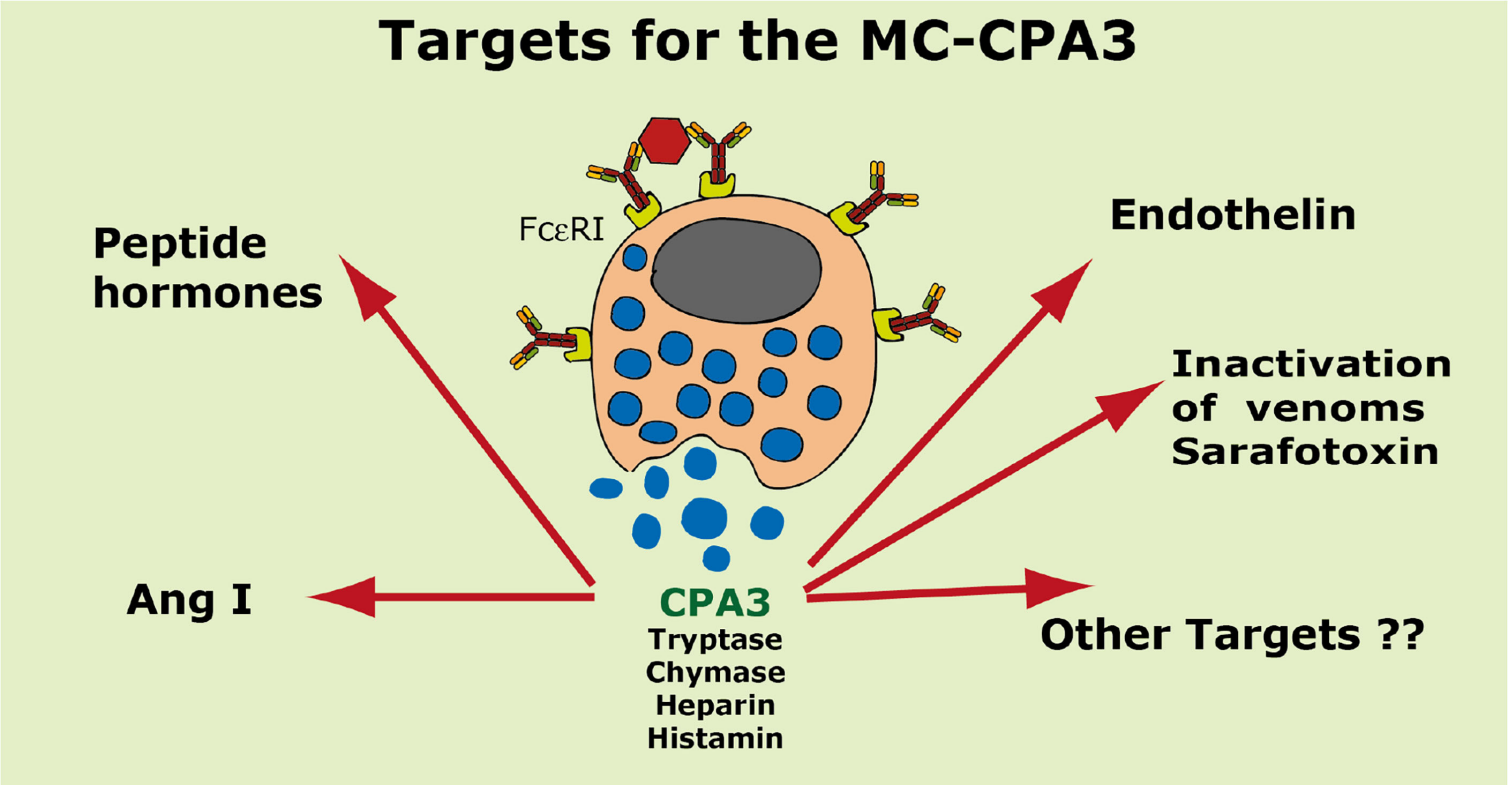
Targets for the human MC-CPA3. A number of potential targets for the human MC carboxypeptidase A3 (CPA3) is summarized.

A summary of the potential targets of the MC CPA3 is found in **Figure 8**.

## *In Vivo* Targets for the Basophil Specific Proteases mMCP-8 and mMCP-11

Two serine proteases have in mouse been found to be expressed preferentially or almost exclusively in basophils, the proteases mMCP-8 and mMCP-11 (39, 40, 62, 119). Numerous attempts using several methods including phage display, chromogenic and recombinant substrates have failed to obtain information concerning the extended specificity of mMCP-8, why we started to wonder if the protease was inactive. Using whole cell extracts and recombinant mouse proteins have later shown that it is active but highly restrictive in its cleavage pattern. Whole cell extract has led to the identification of tubulin as one potential target (119). However, tubulin is most likely not a biologically important target for this enzyme as tubulin is an intracellular protein and mMCP-8 is most likely only located extracellularly after basophil granule release. Two additional potential targets were recently identified by the screening of a panel of mouse cytokines and chemokines. By using recombinant mMCP-8 on a panel of 52 recombinant mouse cytokines and chemokines we identified mouse PDGF-B and MIP-3a as targets (92). However, a screening in the primary amino acid sequence of these three proteins have not resulted in the identification of a consensus sequence for its cleavage preference (92). The three amino acids that form the substrate binding pocket of mMCP-8 indicate that this protease has a strong preference for negatively charged amino acids in the P1 position (**Figure 1**). Mouse and human granzymes B, which are strict asp-ases, have triplets AGR and TGR, respectively. The arginine in this triplet is responsible for the preference for negatively charged residues in the binding pocket. mMCP-8 and rat rMCP-8, -9 and -10 (all members of the M8 subfamily) have ARR in this pocked (25). This is twice the number of positive charge indicating an even stronger affinity for negatively charged amino acids in the P1 position of substrates (**Figure 1B**) (25). However, this preference for negatively charged residues in the cleavable position (P1) has not yet been possible to prove definitely.

Cleavage of PDGF-B may have a functional role in controlling excessive fibrosis during a tick infestation (92). However, the role of MIP-3a cleavage depends on the role this cleavage has on the activity of this chemokine. N or C-terminal cleavage can sometimes increase the activity of a cytokine or chemokine and we still do not know if this N or C-terminal cleavage of mouse MIP-3a results in inactivation or activation of this chemokine.

Interestingly also are other potential effects of mMCP-8 and mMCP-11. Both of them have namely been shown to induce inflammation and influx of inflammatory cells upon injection into the skin of mice indicating a complex role in inflammation (40, 119). The targets for these effects are, however, still not known.

**Figure 9:**
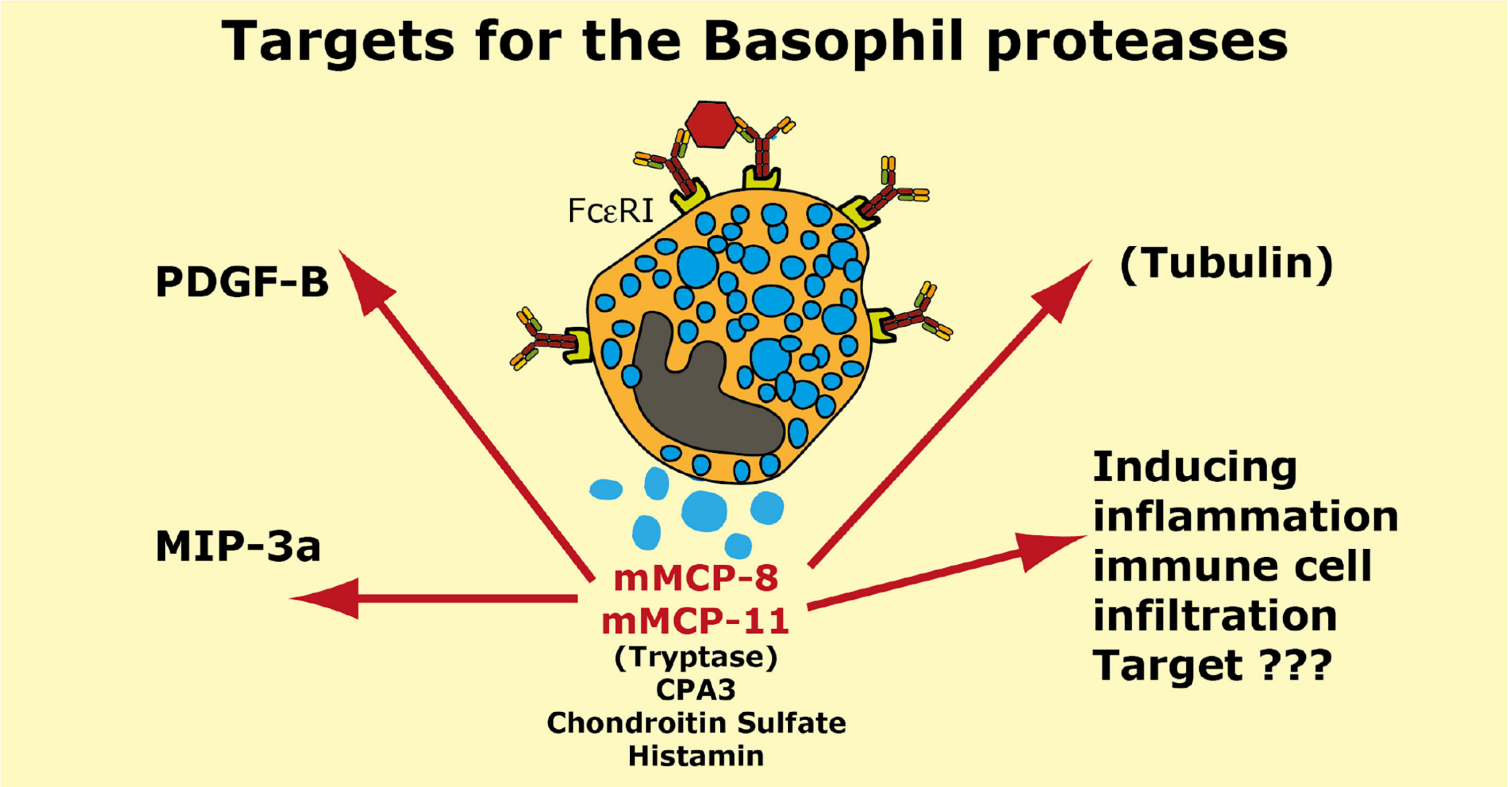
Targets for the mouse basophil proteases mMCP-8 and mMCP-11. A number of potential targets for the mouse basophil proteases mMCP-8 and mMCP-11 is summarized. It is only for mMCP-8 that we so far have any targets identified in mouse PDGF-B, MIP-3a and tubulin. For both mMCP-8 and mMCP-11 we know that they induce inflammation and immune cell infiltration. Human basophils express the MC-tryptase and CPA3. Mouse basophils also express CPA3.

A summary of the potential targets of the basophil specific proteases mMCP-8 and mMCP-11 is found in **Figure 9**.

## Summary

A number of quite diverse physiological effects have been identified for the very abundant granule proteases of MCs. MCs can act both as inflammatory cells by releasing a number of potent physiologically acting substances such as histamine, prostaglandins, leukotrienes and cytokines, but also act in dampening such reactions by selective cleavage of key T_H_2 cytokines. MCs have been shown to produce many of these cytokines and seem now also to be able to trigger keratinocytes, by the action of the tryptase on the protease activated receptor 2 (PAR-2), to produce TSLP. In the same study histamine was able to induce another set of cytokines by these keratinocytes, IL-6, IL-8 and SCF indicating potent effect of both proteases and other granule components in the regulation of cytokines and chemokines by other tissue cells, like the keratinocytes (97).

The MC enzymes can most likely also play an important role in facilitating the influx of inflammatory cells by cleavage of connective tissue components to loosening up the compact connective tissue and also to inhibit coagulation when blood vessels open for influx of antibodies, complement and inflammatory cells. To this should be added the potent effects on numerous venoms from snakes, scorpions, Gila monster and bees (78, 79). The effect on venoms seems to be very complex and involves all three major classes of the mast cell proteases, the chymase, the tryptase and the CPA3. A similar effect may also apply to ectoparasites, by cleavage of their anticoagulants, which are essential for a successful blood meal. Here it is primarily the connective tissue MC chymases that seem to have this role (92). However, the saliva of the ectoparasites is very complex with many different proteins with various biological effects (92). Both tryptase and CPA3 may therefore have effects of other components than only the anticoagulant proteins, which are the prime targets of the connective tissue MC chymases. When it comes to the potential role of the MC proteases in blood pressure regulation we have a similar situation as for the cytokines. One of the MC enzymes, the chymase can generate a blood pressure increasing peptide Ang II, from the precursor Ang I. At the same time another of the MC enzymes, the CPA3, has the opposite effect by inactivating endothelin, the most potent vasoconstrictor known. The question is how these two activities are coordinated during an inflammatory response involving MC activation and degranulation?

The role of the MC proteases in bacterial defense and in autoimmunity has also been controversial. Initially a number of articles appeared in the literature showing potent effects of MCs and also their proteases in the defense against bacterial infection. However, most of these effects did disappear when more stringent MC deficient animal models were used (67). The more stringent models have later shown that MCs do have a role in bacterial clearance, at least in the urinary bladder (69). A number of bacterial virulence factors are most likely also targets for these MC proteases and for the related neutrophil proteases, including N-elastase, proteinase 3 and cathepsin G, however, to our knowledge no direct evidence for such a role *in vivo* for the MC proteases has been documented.

The analysis of the cleavage activity on the anticoagulants from ticks, leeches and mosquitos by a large panel of hematopoietic serine proteases showed a remarkable specificity of these protease. The connective tissue MC proteases efficiently cleaved these anticoagulants whereas the mucosal MC proteases were inactive (92). This despite the fact that they have a relatively similar cleavage specificity indicating that the difference in specificity is highly relevant for their *in vivo* function (26). The connective tissue proteases sit in the skin where they can encounter the anticoagulant proteins from these ectoparasites whereas the mucosal MC proteases are found at other tissue locations where there is little chance to come in contact with these anticoagulants. Evolution has thereby fine-tuned their specificity to fulfill their function to fit their specific *in vivo* location. The very similar pattern in the cleavage products of fibronectin after cleavage with human, macaque and dog MC chymase also indicate conserved targets over relatively large evolutionary timelines. For some of the targets we see a conservation over several hundred million years involving almost all mammals whereas for others the pattern is more complex as seen for the generation of the blood pressure regulating Ang II. A broad analysis involving MC enzyme from all three extant mammalian lineages, monotremes, marsupials and placental mammals, for a larger panel of potential substrates will be needed to fully understand the role of these MC enzymes in MC biology and how conserved these functions have been during the past 200 million years of mammalian evolution.

## Data Availability Statement

The original contributions presented in the study are included in the article/supplementary material. Further inquiries can be directed to the corresponding author.

## Author Contributions

Conceptualization, LH. Writing original draft, LH. Reviewing and editing, SW, SA, and ZF. All authors contributed to the article and approved the submitted version.

## Funding

This study was supported by the Knut and Alice Wallenberg Foundation (KAW 2017-0022) to LH and SW, by Konsul Berghs Stiftelse to SA and by the Swedish Heart and Lung Foundation to SW.

## Conflict of Interest

The authors declare that the research was conducted in the absence of any commercial or financial relationships that could be construed as a potential conflict of interest.

## Publisher’s Note

All claims expressed in this article are solely those of the authors and do not necessarily represent those of their affiliated organizations, or those of the publisher, the editors and the reviewers. Any product that may be evaluated in this article, or claim that may be made by its manufacturer, is not guaranteed or endorsed by the publisher.

## Abbreviations

MC, mast cell; MMC, mucosal mast cell; CTMC, connective tissue mast cell; Ang II, angiotensin II; CPA3, Carboxypeptidase A3; mMCP-1, mouse mast cell protease 1; rMCP-1, rat mast cell protease 1; MC_T,_ mast cell expressing only the tryptase; MC_TC_ mast cell expressing both the chymase and the tryptase; TSLP, thymic stromal lymphopoietin; AT1, angiotensin receptor 1; ACE, angiotensin converting enzyme; NK cell, natural killer cell; Vch, vascular chymase; TAP, tick anticoagulant protein.
